# On the Evidence of the European Bee-Eater (*Merops apiaster*) as a Predator of the Yellow-Legged Hornet (*Vespa velutina*) and Its Possible Contribution as a Biocontrol Agent

**DOI:** 10.3390/ani13121906

**Published:** 2023-06-07

**Authors:** Nuno Onofre, Maria Inês Portugal e Castro, Anabela Nave, Irene San Payo Cadima, Maria Ferreira, Joana Godinho

**Affiliations:** 1 National Institute for Agrarian and Veterinary Research I.P., Av. da República, Quinta do Marquês, 2780-157 Oeiras, Portugal; ines.portugal@iniav.pt (M.I.P.e.C.); anabela.nave@iniav.pt (A.N.);; 2 CITAB—Centre for the Research and Technology of Agro-Environmental and Biological Sciences, University of Trás-os-Montes and Alto Douro, Quinta de Prados, 5000-801 Vila Real, Portugal

**Keywords:** Yellow-legged Hornet, European Bee-eater, control measures, limiting factors, predation, Iberian Peninsula, alopatry

## Abstract

**Simple Summary:**

The Yellow-legged Hornet is an exotic and invasive insect which, having entered Europe through France in 2004, has now spread across many countries of the continent. It is a threat to biodiversity and causes significant damage to beekeeping. Control measures have not been able to stop the expansion of this hornet, so all means of fight are important, including natural predators. We studied the diet of the European Bee-eater to understand its role and predation intensity on the Yellow-legged Hornet. We found several hornet remains in some locations of Central Portugal. Although the importance of this predation remains to be determined, Bee-eater could be one more agent in the fight against the biological control of this pest, even though the two species do not coexist in much of the Iberian Peninsula. However, in a large area of the rest of Europe, it is predictable that the distribution range of the two species will overlap to a greater extent and the Bee-eater may play a greater role as a biological control agent.

**Abstract:**

The Yellow-legged Hornet (*Vespa velutina nigrithorax*) (YLH) is an invasive insect that arrived in Europe in 2004 and is now spread across nine countries. It is a threat to the native entomofauna and harmful to beekeeping and agriculture, as it is a ravenous predator of the European Honey Bee (*Apis mellifera*) and other pollinating species. Its expansion has been unstoppable and all resources are needed to fight against it, including native vertebrate predators. Among these, the European Bee-eater (*Merops apiaster*) (EBE) is a potential one, but little is known about its predation on YLH. In a study carried out in Portugal, remains of YHL were detected in EBE nesting sites, which, to the best of our knowledge, is the first such report. This means that this bird could be one more agent in the biological control of this pest (although research on predation intensity is still needed), in conjunction with other natural predators and other strategies. In the Iberian Peninsula, both species are allopatric in vast regions, so the role of EBE may be more limited. However, in the rest of Europe, at a country or continent scale, the scenario may be different and sympatry may occur to a greater extent.

## 1. Introduction

The Yellow-legged Hornet (*Vespa velutina*) (YLH), originates from Southern, Eastern and South-eastern Asia [1,2], is an insect that has become invasive in the European continent and is very concerning in terms of economic and biodiversity damages, and ecosystem sustainability. It is represented by the subspecies *V. v. nigrithorax* which is native to Nepal, India, Bhutan, Vietnam and China [2]. The YLH first arrived in Europe via France around 2004 (supposedly from 1 or very few founder females that came in Chinese pots imported by a bonsai seller), and then expanded to several other European countries, namely Spain in 2010, Portugal and Belgium in 2011, Italy in 2012, Germany in 2014, UK in 2016, Netherlands and Switzerland in 2017 and more recently Luxembourg in 2020 [3,4,5].

In Europe the YLH has been found to cause much negative economic impacts on beekeeping (predation on honey bees and losses of swarms, with consequent damages in the production of honey and other bee products), and on agriculture (decline of crops pollination, and damage in orcharding and other fruit plantations) [6,7,8,9,10]. Impacts on biodiversity (predation over many native insect species, including other Apidae, Vespidae and insect pollinators; capture and collateral destruction of dozens of non-target native insect species in non-selective traps), and possibly on ecosystem functions and services (e.g., pollination declining of wild and domestic plants and changes in trophic chains) are also referred [6,8,9,10,11,12].

Several researchers claim that it is now impossible or nearly impossible to eradicate the YLH [7,9,13], which will continue to spread in the countries where it is already present [10,14,15], and that in the long term or even in a few years it will very likely spread over most of the rest of Central, Eastern and Southeastern Europe [1,13,16,17,18,19]. Even with the application of the current methods of destruction of this exotic hornet by the authorities and beekeepers (surveillance, use of baited traps, location and destruction of nests), the spread has been unstoppable [9,20,21,22]. The use of biological methods has been limited to the use of parasitic insects, nematodes, entomopathogenic fungi and acari [22]. Also, according to Turchi & Derijard [22], these authors, so far only the first two methods have proven to infect YLH, although there is much hope that the other two are likely to infect YLH too. Nevertheless, all these methods are still in the experimental phase [22]. Thus, all resources are needed to fight against this pest in order to reduce the density of its populations in areas it has invaded and occupied, and this should include potential natural predators.

Several species of wild animals in Europe are known to feed on wasps and some even capture the large European Hornet (*Vespa crabro*). However, very often, published studies on the feeding habits of insect predators do not discriminate prey at the species or genus level, and sometimes not even at the family level. So, references to the capture of wasps of the genus *Vespa* by a specific predator are not very common.

Wasp predators are mostly birds, but there are also references to several mammal species preying on or opportunistically consuming wasps [7,23,24]. For example, in Europe, the European Badger (*Meles meles*) is known to dig the secondary nests of social wasps in the soil, eating larvae, adults, combs and the envelope of the nests [25,26], in summer or early autumn [27]. The Badger is probably the main wasp predator among mammals [25]. According to Edwards [26], moles (*Talpa* spp.) also destroy some wasp nests, although in small numbers, and some species of mice, such as the Wood Mouse (*Apodemus sylvaticus*) or the House Mouse (*Mus musculus*) may also eat or destroy wasp queens existing in the soil, when they are hibernating. In turn, van Bergen [28] detected European Pine Marten (*Martes martes*) destroying a wasp nest, possibly of *Dolichovespula sylvestris*, and Spradbery [25] also mentions other Mustelidae-Stoat (*Mustela erminea*) and Least Weasel (*M. nivalis*), which, as well as the Wood Mouse, disrupt colonies that are still incipient in mouse burrows in the ground.

Predation or destruction of European Hornet nests by the European Badger and Red Fox (*Vulpes vulpes*), and probably also by the Pine Marten or Beech Marten (*Martes foina*), was recorded by Nadolski [29]. The Common Wall Gecko (*Tarentola mauritanica*) has also been observed successfully hunting the European Hornet in Sardinia, Italy [30]. However, since the vast majority of YLH nests are located above ground, mostly in trees [31,32,33,34,35], the impact of mammals on this hornet is likely much smaller.

As far as birds are concerned, there is abundant scientific documentation attesting their predation on a large number of species of Hymenoptera and, among these, on Vespidae. The vast majority of species that consume wasps are generalists-insectivores or omnivores-, capturing them at random, but a very restricted number of species are specialized in Hymenoptera or even in Vespidae.

Regarding the bird species known to feed on the European Hornet, we found references to the European Bee-eater (*Merops apiaster*) (EBE) [36,37,38,39,40,41], European Honey Buzzard (*Pernis apivorus)* [28,42,43], Iberian Grey Shrike (*Lanius meridionalis*) [44], Red-backed Shrike (*Lanius collurio*) [45], Eurasian Jay (*Garrulus glandarius*) (Stachanoff cit. in Spradbery [25], and Eurasian Magpie (*Pica pica*) [46]. Since these birds are able to catch hornets such as the European Hornet, they are also likely potential predators of the YLH, as it is slightly smaller and its nests are mostly out in the open, in trees and bushes [31,32,33,34,35].

**Figure 1:**
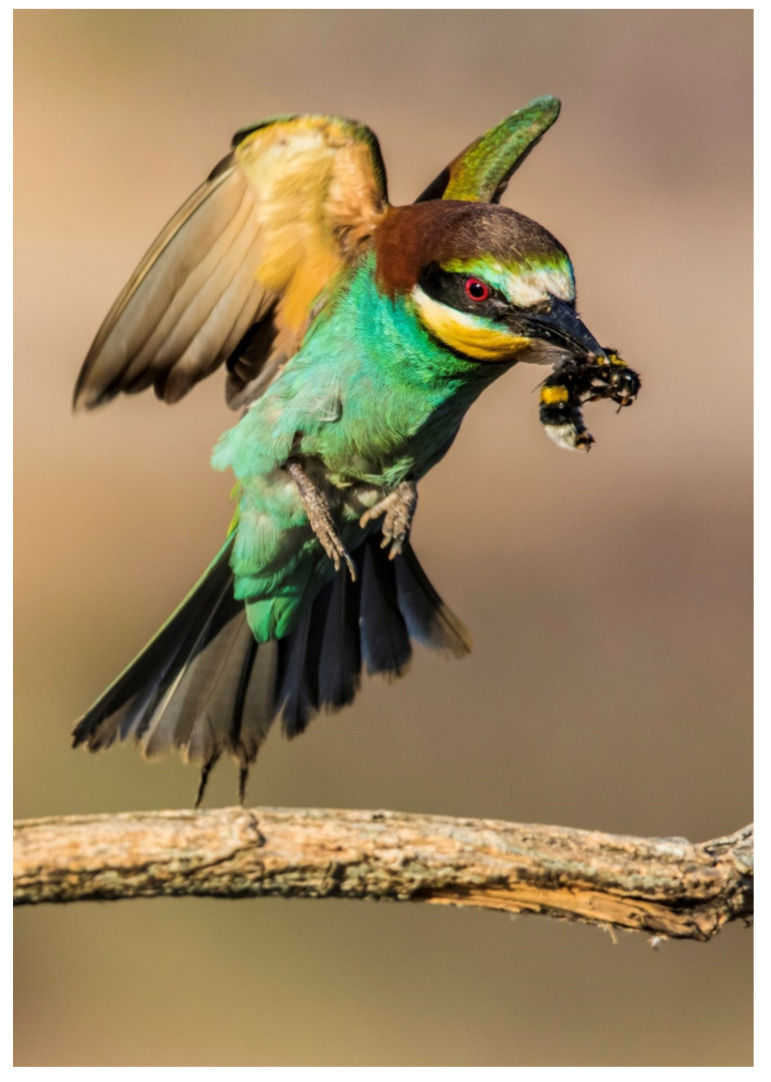
European Bee-eater (*Merops apiaster*) (Photo credit: José Freitas).

The Iberian Grey Shrike [47], the European Honey Buzzard [48,49,50,51] and the EBE [52] are among the bird species for which predation on the YLH has already been confirmed. Mollet & de la Torre [31], Villemant et al. [53] and Rome & Villemant [15] (authors from which most subsequent articles are based when the potential predators of the YLH are mentioned, although they do not refer to dates, locations or eventual original sources of information), also report that woodpeckers (e.g., *Picus* sp., *Dryobates minor*), Magpie (*Pica pica*) and tits such as the Eurasian Blue Tit (*Cyanistes caeruleus*) were observed in late autumn/early winter pecking the nest envelope to consume the last remaining individuals, larvae or torpid adults, of the respective declining colonies. Additionally, Bunker [5], quoting F. Muller (pers. comm.), states that “*There have been rare records of more specialist enemies such as the European Honey Buzzard (*Pernis apivorus*) and the European Bee-eater (Merops apiaster), but these have a negligible impact on the population*”. However, here, again, information is not accurate for the reasons mentioned immediately above and because so far we have found at least four articles that accurately refer or document YLH predation by the European Honey Buzzard—two of them published in scientific journals [49,50]. With the exception of our own work [52], there is a lack of information on EBE predation on this pest (Figure 1).

However, the opinion concerning predation importance of the Honey Buzzard is not consensual, namely from the Spanish researchers who found YLH breeding combs in buzzard nests. In fact, Macià et al. [49] refer that this bird of prey should be considered as a potential biocontrol agent since it is capable of destroying active nests of YLH, and, at the same time, Rebollo et al. [50] report that the Honey Buzzard population in Galicia (the largest and second densest one in Spain [54]) could destroy thousands of YLH nests every year, that is equivalent to the number of nests destroyed by the authorities control teams (S. Rebollo cit. in Anonymous [55]).

The contribution of each of the species mentioned above in the natural limitation of the expansion of YLH, as well as of other birds that consume Vespidae, should not be significant. However, EBE and Honey Buzzard may be the exception, as they are specialists, or more specialized, in Hymenoptera and Vespidae, respectively [42,56,57,58,59,60]. Therefore, these ones are the most important potential enemies of YLH among wild vertebrates, and so they have the potential to contribute to the biological control of this invasive pest in a more significant way. For this reason, and because there was little information about the predation by these two species on the YLH, mainly in what concerns the EBE and for which, till then, nothing had been published on this matter, in 2021 the National Institute for Agrarian and Veterinary Research (INIAV), in Oeiras, Portugal, started a study on the diet of this latter species to assess the relative importance of its predation on YLH.

The EBE’s diet in Europe is largely composed of European Honeybee (*Apis mellifera*) and bumblebees (*Bombus* sp.), with prey captured usually in the air [59]. The European Hornet is also part of its diet, but it is an uncommon prey species [37,38,40,61]. In a set of 38 studies on the EBE diet, Vespidae represented, on average, around 8–9% of the total prey, only reaching 30–40% in 4 of these studies [59], whereas in Spain the percentages of Vespids do not go beyond 2% [62,63,64]. In Portugal, according to Costa et al. [65], the composition of the EBE’s diet varies according to the country’s regions, but is mainly composed of Hymenoptera (the majority) and Coleoptera, which totalize approximately 90% of the items consumed in the breeding season (adults and nestlings). Concerning only the nestlings, Hymenoptera constitutes 68% to 85% of their diet wherein-European bees corresponding to 26–47%, wasps ~3–33%, and other Hymenoptera 21–30% of the diet [65].

However, the importance of predation also depends on other factors, such as the geographic coexistence between both species, predator and prey; in addition, although the potential area of occurrence of YLH in Europe (see Verdasca et al. [19] or GBIF Secretariat [66], among others), coincides very reasonably with the distribution range of the European Honey Buzzard [67,68], the same does not seem to happen with the EBE, at least in the present and in certain regions of Europe [68,69].

## 2. Materials and Methods

**Figure 2:**
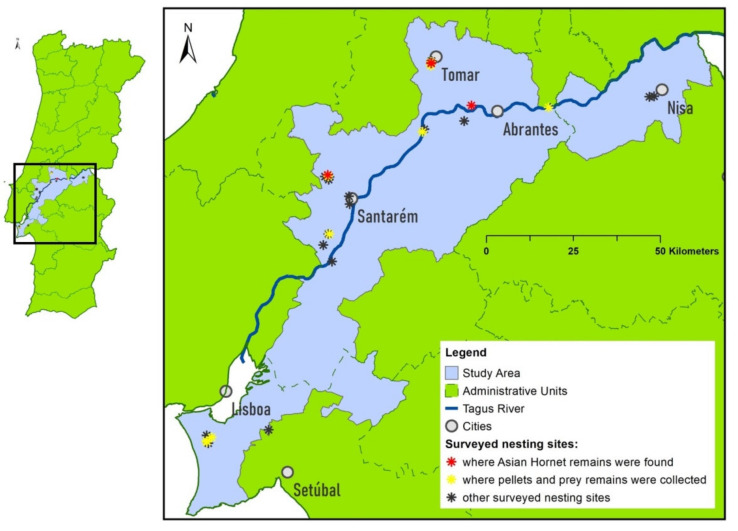
Study area and Bee-eater nesting sites locations [Because the scale of the figure is very small, several locations/asterisks are overlapping; Administrative Units based on Oficial Cartography for Portugal (CAOP) from D.G.T. (Direção Geral do Território)].

The occurrence areas of the YLH and the EBE in Portugal are somewhat disjointed [70,71], so the study area was located in the region where these areas overlap. The study area comprised the counties of the district of Setúbal, that are located on the left bank of the Tagus River in front of the city of Lisbon, and the counties of the districts of Santarém and Portalegre bordering the same river (Figure 2).

Search for nesting sites started in March of 2021, with many nesting sites located after information from fellow ornithologists. Pellets and prey remains (complete insects or parts thereof) were collected from May to September of the same year, and once a month, usually in the last week.

**Figure 3:**
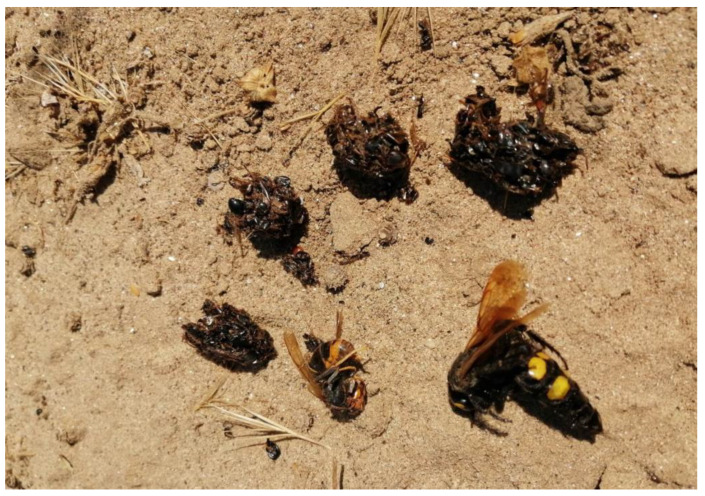
Pellets and whole prey individuals [Yellow-legged Hornet (*Vespa velutina*) (bottom left) and a Mammoth Wasp (*Megascolia maculata*) (bottom right)] of European Bee-eater.

In nesting sites where it was difficult to collect food material, due to the inaccessibility of the nests or the absence of perches, artificial perches (dry branches) were placed nearby in order to increase pellet sample sizes, which was successfully achieved. Most of the material was collected under perches, but some was also collected inside nest tunnels (in this case, mainly remains of crumbled pellets, but also of some preys), or on the ground below its entrances after fledging. After collection, each pellet was stored in an individual bag and, together with the remains of prey found (Figure 3), stored in a larger bag corresponding to the respective nesting site. This bag was later stored in a freezer until it was analyzed.

Since bee-eaters rarely take again a fallen prey that was carried in the beak (Koenig & Ursprung cit. in Bastian & Bastian [59], it is rather easy to find a fair number of whole insects captured by these birds near their nests and roosting sites.

The pellets were kept for further analysis by molecular genetic methods in order to determine the frequency of occurrence of YLH as a food item and its importance in the EBE’s diet, and therefore the impact that this bird may have in limiting this very harmful exotic hornet to the beekeeping activity.

At present, only a first identification of prey remains collected in 2021 has been conducted. Thus, only preliminary results on prey captured by this bee-eater are presented.

Prey remains were identified with the aid of a 7-45x Euromex ZE.1654, Stand no. 1740-H, insect field guides (e.g., Chinery [72]) and, whenever necessary, with the expertise of fellow entomologists at INIAV. They were processed and identified considering the site and date they were collected.

## 3. Results

At present, only a first identification of prey remains collected in 2021 has been conducted. Thus, only preliminary results on prey captured by this bee-eater are presented here.

Most of the pellets and prey remains were collected in June and July, when the birds were feeding their young. Few of them were collected in May, when the birds were incubating their eggs, as well as in August/September when juveniles and adults forage far from nesting sites and spend little time there and begin their autumnal migration.

Out of a total of 30 nesting sites located (Figure 2), only in 13 food material was collected. This is because some colonies did not become active that year, or because nesting sites with few nests were abandoned due to breeding failure early in the season, or because they were completely inaccessible.

Table 1 shows the set of prey species identified among the remains of insects found on the ground below the perches and in the nest tunnels of the EBE nesting sites surveyed.

One of the main objectives of the present study was to study YLH as Bee-eater prey. To this end, we found prey remains parts of 5 specimens of YLH, collected in 4 different nesting sites (Figure 2). Except for the two nearby places in the region of Tomar, the other places where the YLH was found were more than 20 km (Abrantes region) and 50 km (Santarém region) away from the first two. The parts of the hornet that were found are a 1st abdominal segment, a complete individual, an individual without a head, an isolated head and, finally, a mandible and two abdominal segments (1st and 2nd).

In addition to the YLH, a complete specimen of European Hornet and 16 specimens of the large Mammoth Wasp (*Megascolia maculata*) were also found. Many also intact (Table 1). The latter wasp and bumblebees (*Bombus* sp.) were the insects most frequently found among prey remains after the European Honey Bee (*Apis mellifera*) and, so far, also after some smaller wasp species such as *Polistes* sp. or *Vespula* sp.

Among the remains of prey, the Hymenoptera seems to be the most important prey group of the studied bee-eaters, followed by the Coleoptera, and within this order, the genera Cetonia and, particularly, Protaetia of the Scarabaeidae family, are the most captured beetles.

## 4. Discussion

In the first place, it is important to point out those methods of assessment of diet based on prey remains are subject to several biases. For this very reason, it is possible that the data presented in Table 1 are somewhat biased. One of the main reasons has to do with the fact that the remains of larger and/or more colorful prey, whether made up of whole specimens or their parts, tend to be more easily detected and collected by the members of the study team. For this reason, it is possible that the proportion of Mammoth Wasp or beetles of the Cetoniinae subfamily and others maybe overrepresented and that of honey bees and smaller Apidae and Vespidae underrepresented. On the other hand, it is also to be expected that the remains of more palatable prey species, involuntarily dropped to the ground by the bee-eaters, maybe preferentially collected by other wild animals, vertebrates or invertebrates. Still on the subject of palatability, the high relative number of Mammoth Wasps found could also be related to the fact that, although it could be a relatively easy prey to capture, it could end up not being very palatable. The analysis of the pellets may help to clarify the composition of the EBE diet more closely.

The findings of several Yellow-legged Hornet individuals caught by the Bee-eater in Central Portugal [52], provide evidence that this pest is also a prey of this bird in Europe. To understand the importance of this predatory activity is outside the scope of the present work but will be followed up on in due course. The capture of the YLH was expected, as the EBE has always hunted wasps and among these, although in small numbers, the great European Hornet [37,38,41,61,63]-an even larger hornet -, and also because this exotic hornet is expanding territorially [73]. The data presented here are still very incipient and caution is needed in extrapolating from them. In any case, it could be that the greater number of YLH individuals found among prey remains than that of European Hornet, may indicate either a greater availability of the first species or that it is easier to capture. In both cases, this could suggest that EBE may have a role in the biological control of YLH.

With regard to the specific effectiveness of the EBE in capturing and feeding on YLH, it is important to know how much this hornet contributes to the diet of the insectivorous bird. Work is currently underway to further understand this predation potential and this will require another year of collecting material on the ground. Yet, no matter how much YLH the EBE captures, this effort will only be effective in areas where both species, prey and predator, cohabit. Indeed, contrary to what happens with the European Honey-buzzard, a predator specialized in Vespidae and which in France, Spain and Portugal has already been confirmed to feed on combs from YLH nests [48,49,50,51], and whose distribution ranges in Europe, both in the present and in future predictions, seem to overlap reasonably [17,19,67,68,69], the same does not seem to occur always with EBE.

**Figure 4:**
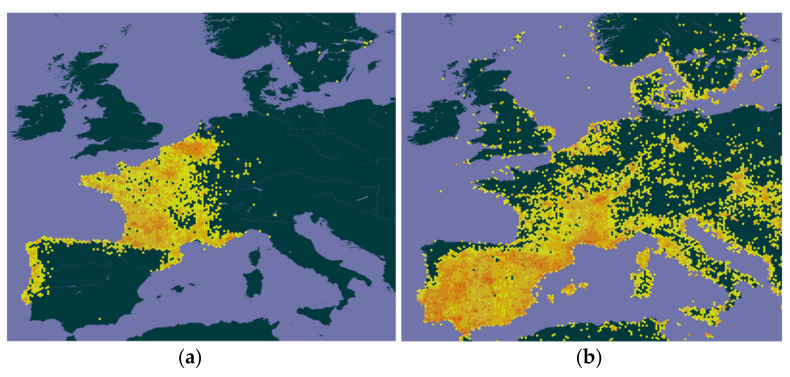
Distribution of Yellow-legged Hornet (**a**) and Bee-eater (**b**) records in Europe according to GBIF Secretariat [66,69].

The two species, the YLH and the EBE, seem to have somewhat different climate niches, with the YLH preferring wetter and fairly cooler regions [10,19,21,33,74,75], while the EBE, as a thermophilic species, prefers regions of greater aridity, that is, hotter and drier [57,59,76,77,78,79,80,81]. For this reason, on a country-wide scale, the current area of sympatry between the two species does not appear to be particularly large, namely in the Iberian Peninsula (IP), as illustrated in Figure 4, which, despite some flaws (e.g., in England), reasonably reflects the distribution of the invasive wasp [19,73,82] and the EBE [68] in Europe. Presently, in Portugal, the overlap between the areas of occurrence of the two species is not very large, as already mentioned, and in Spain the disjunction seems to be much more evident (cf. Figure 4). Indeed, most of risk, adequacy or general favorability models in general, which address the expansion of the YLH and cover Portugal, Spain or IP as a whole [4,13,16,17,19,33,73,74], suggest that almost half of the Portuguese territory (i.e., most of the provinces of Alentejo and Algarve, in the south, and some parts of the east, north of the Tagus River), and much of Spanish territory (e.g., the autonomous communities of Extremadura, Andalusia, Castilla-La Mancha and Murcia, located in the center and south of this country) [21,33], will have low or medium-low suitability for YLH. Paradoxically, these regions, where the probability of expansion or occurrence of the YLH is low, very low or even null due to climatic inadequacy [13,17,19,33,73,74], are exactly those where most of the EBE populations are traditionally located and where its populations are strongest [71,83,84,85,86,87]. It is true that in the future, in the medium and long term (2030s to 2100s), the predictions point to an increase, not very accentuated, of the climate suitability for YLH in these regions of the IP (smaller in the referred areas of Spain) [16,17,20], and a slight expansion of the EBE northwards into the Eurosiberian region of the IP by 2040, 2050 and 2100 [81,82,83,84,85,86,87,88], an expansion that has been observed since the late 1990s [86,87]. However, it does not seem that there will be a significant increase in the overlapping of the ranges of the two species.

With regard to France, Figure 4 also shows that there seems to be some spatial segregation between YLH and EBE, except in the south in the regions of Occitania and Provence-Alpes-Côte d’Azur and in Rhône-Alpes, where coexistence it is more consistent. However, contrary to what happens in IP, both the YLH and the EBE are present in almost all the France, with the hornet occurring in all but one of its 96 departments (i.e., except Haut-Rhin) [15,89] and the EBE being present or seen in almost all French departments, breeding in all the 13 major administrative regions [68,90,91], although significantly more rare and irregular in the north and northwest of the country, in the Center region and the central Massif [59,92], as evidenced, moreover, in the cartography [68,90,91]. However, there is a greater spatial coincidence at the regional scale in France than in IP, or a greater probability of joint occurrence of the two species, and this overlap in YLH and EBE distribution ranges is expected to increase further in the future due to climate change. Indeed, Stiels et al. [81] forecast a significant increase in the range and on the probability of occurrence of the EBE in a large part of France in 2050, which is predicted to be medium to high in much of its territory, at the same time that the climate suitability models for the YLH, either for the short term [13,17,19], or for the medium-long term-2030 to 2100 [16,17,20], forecast equal or greater likelihood of YLH occurrence in almost all of France. That is, in the medium-long term, the probability of coexistence of the two species in France at the local scale will be much greater and greater than in the IP.

In the rest of Europe, the same climate suitability models point, in general, to an expansion of YLH in a large number of countries, such as in Germany, Belgium, the Netherlands, Switzerland and Luxembourg, the United Kingdom and Italy where it has already arrived or settled [15,82] or in the east and southeast of this continent, where it is expected to settle in the medium-long term [13,16,17,19,20]. In turn, the EBE, which since the 1980s and 1990s has been expanding to the north and its populations have been growing in several European countries [59,79], is present in all those countries, although in most of them their populations are in generally still small or nesting is still irregular (e.g., UK, The Netherlands, Luxembourg or Belgium) [59,68,93]. This northward expansion across the European continent, including France, has been largely attributed to climate changes and an increase in food availability as a result of these [59,77,78,79,80,81,82,83,84,85,86,87,88,89,90]. In Germany, where the population has grown unparalleled in Central Europe, the EBE is present in almost all country and federal states, i.e., with the exception of the 3 urban states of Berlin, Hamburg and Bremen [90,93]. In Italy, as is typical in a Mediterranean European country, the EBE population is even stronger and is distributed over most of the territory [59,68]. Currently, EBE populations in countries of Central and Northern Europe are far from having such a wide national distribution as in countries of Southern and Southeast Europe [68], but predictions for the future (2050) point to a significant increase in the probability of occurrence of this bird in Central Europe, Northern Europe and Eastern Europe [1,81], and consequently in its territorial and population expansion. That is, the probability of spatial coexistence of the two species, hornet and Bee-eater, in these regions is once again quite reasonable, at least on a regional scale, and, once again, higher than could happen in IP. On the other hand, since the availability of large insects is an important factor for the establishment of the EBE in new territories [90], expansion of YLH may contribute the expansion of the bird. In the eventuality, as is expected [19], that the YLH expands further eastwards, especially towards Southeastern Europe, where climate suitability for this insect is expected to be largely medium to high [17,19]. If so, the possibility of territorial coexistence of both species in this part of the European continent is quite reasonable, since the EBE is currently widely distributed across the territories of these countries [59,68], and tends to be even more common in the future [81].

However, despite the existence of correspondence between their distribution ranges on a continental or national scale, the potential contribution of the EBE in the biological control of YLH will only be effective when both species coexist locally, which depends, among other aspects, on the availability of nesting habitat for the EBE. During the nesting season, the geographic distribution and density of the EBE depends on the availability of soft geological substrates, such as sand, sandstone, clay, gypsum or loess, and, as a rule, on the existence of steep walls in these substrates where the species can dig galleries to build their nests [59,87]. Traditionally, this occurs mainly on the banks of rivers and lakes [59,78], which, according to some authors, the YLH seems to take advantage to expand, particularly the river valleys [32,35], or build large number of colonies, among other habitats [94]. However, nowadays, as happens mainly in Central Europe, EBE colonies are common in sand and gravel pits, quarries, open pit mines, mining subsidence areas, terraces of olive groves and vineyards, railway and road slopes, among other anthropogenic habitats [59,87], which could contribute to a greater territorial dispersion of the bird. On the other hand, the role of the EBE in YLH control will also be conditioned by its quite small home range, which rarely exceeds 1 km from the nesting site during the nestling period, with most foraging activity limited to the first 700 m [59]. Only after fledging and until departure for Africa does the foraging distances increase, reaching extents as far as 15-17 km [59]. From this time onwards and when they migrate southwards towards the wintering grounds of Africa (mainly from August to September/October, but also in November; Bastian & Bastian [59]), EBE predation on the YLH could even be as important as during the nesting season, if not even more. Indeed, this is exactly the time when the YLH colonies reach their maximum growth and activity (from September onwards, but mainly in October and November, concerning the number of workers and the emergence and mating flights of sexual adults [21,32,95]), thus, coinciding with the time when the birds are no longer “attached” to the nest site and form flocks of some dozen individuals (20–40 birds) [59], which can move anywhere searching for food and opportunistically taking advantage of this increased availability of YLH.

Finally, it should be said that the most important growth of YLH colonies in France seems to occur from August/September to December, with maximum values in October/November [95], while EBE remains in Europe between April and September, rarely in October and November [57,59,85]. Incidentally, according to the aforementioned authors, during the month of September EBE is in full migration in European countries. This means that in France, and possibly the rest of Western Europe (cf. UN [1]), the EBE will “miss” the two months when populations of YLH, workers and sexual individuals such as founding queens are most abundant. In Portugal, however, the YLH biological cycle seems to start almost a month earlier than in France (cf. Rome et al. [95]; Nave et al. [8]). In 2016–2017, July and August were the months in which the most hornets were captured in Portugal, followed by September, October and June in terms of abundance [96]. June/July to September was the period in which there was greater predation pressure from hornets on the bees in the hives [97]. At the same time, the biological cycle of YLH in Galicia, Spain is similar [97]. That is, at least in Portugal and Spain (and perhaps also in the rest of Southern Europe (cf. UN [1]), the temporal overlap between the stay of the EBE and the period of greatest abundance of YLH is quite large, apparently greater than in France and Western Europe.

## 5. Conclusions

It is confirmed that the European Bee-eater captures the Yellow-legged Hornet and, although data are still scarce, this does not seem to be uncommon. This is not surprising as Hymenoptera traditionally make up a large part of its diet. However, the real importance of this predation remains to be assessed, in order to have an approximation to its contribution in the biological control of this pest, which considerably affects beekeeping and biodiversity.

In the Iberian Peninsula, the role of the EBE is limited by the relatively small overlapping of their distribution ranges (in vast regions where there are the most numerous and widespread populations of the EBE the YLH is not expected to reach there and in most of those where it is already invaded the first one does not occur), an aspect that does not seem to happen in the Western and Central Europe, despite the current lower EBE populations in this regions.

## Figures and Tables

**Table 1:** Prey *taxa* found among remains under perches and nest tunnels of the European Bee-eater at the study area in 2021.

Prey *taxa*		Absolute Frequency (No.)	Relative Frequency in Percentage (%)
HEMIPTERA		4	3.1
	Cicadelidae	2	
	Cicadellidae und. *	1	0.8
	Hemiptera und.	1	0.8
DIPTERA		1	0.8
	*Volucella zonaria*	1	0.8
HYMENOPTERA		91	71.1
	Apidae	43	
	*Apis mellifera*	31	24.2
	*Bombus* sp.	8	6.3
	*Melecta* sp.	1	0.8
	*Xylocopa violacea*	1	0.8
	Apidae und (not *Apis* or *Bombus*)	2	1.6
	Crabronidae	1	0.8
	*Cerceris* sp.	1	0.8
	Scoliidae	15	11.7
	*Megascolia maculata*	16	12.5
	Vespidae	28	
	*Polistes gallicus*	8	6.3
	*Polistes* sp.	5	3.9
	*Vespa crabro*	1	0.8
	*Vespa velutina*	5	3.9
	*Vespula germanica*	5	3.9
	*Vespula* sp.	3	2.3
	*Dolichovespula/Vespula*	1	0.8
	Apocrita und.	4	3.1
COLEOPTERA		32	25.0
	Staphylinidae	1	
	Staphylinidae und.	1	0.8
	Geotrupidae	1	
	Geotrupidae und.	1	0.8
	Scarabaeidae	23	
	*Cetonia* sp.	3	2.3
	*Protaetia* sp.	14	10.9
	*Trichius* sp.	1	0.8
	*Cetonia*/*Protaetia*	5	3.9
	Buprestidae	1	
	*Buprestis* sp.	1	0.8
	Tenebrionidae	1	
	*Blaps* sp.	1	0.8
	Curculionidae	1	
	*Rhynchophorus ferrugineus*	1	0.8
	Coleoptera und.	4	3.1
Total number of prey		128	

* und = undetermined.

## Data Availability

Not applicable.
